# Novel Natural Product-like Caged Xanthones Bearing a Carbamate Moiety Exhibit Antitumor Potency and Anti-Angiogenesis Activity *In vivo*

**DOI:** 10.1038/srep35771

**Published:** 2016-10-21

**Authors:** Xiaoli Xu, Yue Wu, Mingyang Hu, Xiang Li, Qichao Bao, Jinlei Bian, Qidong You, Xiaojin Zhang

**Affiliations:** 1 State Key Laboratory of Natural Medicines and Jiangsu Key Laboratory of Drug Design and Optimization, China Pharmaceutical University, Nanjing, 210009, China; 2 Department of Organic Chemistry, School of Science, China Pharmaceutical University, Nanjing, 210009, China

## Abstract

**DDO-6101**, a simplified structure obtained from the *Garcinia* natural product (NP) gambogic acid (GA), has been previously shown to possess high cytotoxicity to a variety of human tumour cell lines. To improve its physicochemical properties and *in vivo* cytotoxic potency, a series of novel carbamate-bearing derivatives based on **DDO-6101** was synthesized and characterized. The structural modifications revealed that the presence of a carbamate moiety was useful for obtaining comparable cytotoxicity and improved aqueous solubility and permeability. **8n**, which contains a bipiperidine carbamate moiety, displayed better drug properties and potential in *in vivo* antitumor activity. In addition, an antitumor mechanistic study suggested that **8n** (**DDO-6337**) inhibited the ATPase activity of Hsp90 (Heat shock protein 90), leading to the inhibition of HIF-1a and ultimately contributing to its anti-angiogenesis and antitumor properties.

Natural products (NPs) have a unique diversity of structures and complexity. They have played and continued to play important roles in the discovery of drugs that are used to treat various diseases. In the clinic, approximately half of the current anticancer agents are NPs or were inspired by NPs^1^,^2^,^3^. Despite their limitations, including poor solubility, undesirable pharmacokinetics and associated toxicity, NPs may still provide core scaffolds with specific stereochemistry. In many cases, to gain new physicochemical properties, improve biological effects, have fewer side effects and increase their drug properties, structural modifications of NPs are necessary, thereby inspiring the whole pharmaceutical industry^4^.

*Garcinia hanburyi* (*G*. *hanburyi*), an anti-inflammatory and hemostatic traditional herbal medicine from Southeast Asia, has received substantial attention from medicinal chemists for centuries. The major bioactive constituents of the gamboges resin secreted by this tropical plant all have a unique scaffold, including a 4-oxa-tricyclo[4.5.1.0^3^,^7^]decan-2-one structure^5^,^6^,^7^. Among these constituents, gambogic acid (GA) (Fig. 1) is considered to be the compound with the greatest curative effect, and it has been positively identified as a potential and promising antitumor agent in recent decades^8^. It is exciting that phase IIa clinical trials of GA in cancer patients were recently completed in China^9^. GA has been shown to induce apoptosis^10^, inhibit proliferation^11^, repress adhesion and metastasis^12^, regulate the cell cycle^13^, and reverse multidrug resistance of cancer cells^14^; GA has also been shown to possess anti-angiogenic activities^15^. Similar to many multi-target NPs, GA also has multiple targets, such as Hsp90, the bcl-2 pathway, and the transferrin receptor in tumour cells^12^,^15^,^16^,^17^,^18^,^19^,^20^,^21^. However, its direct molecular target remains uncertain. Unfortunately, there are some disadvantages to the use of GA, including poor physicochemical properties, such as low aqueous solubility and low bioavailability, and the availability of the gamboges resin^22^. Substantial effort in medicinal chemistry is essential and significant to improving the drug properties of GA and other caged xanthones.

In recent years, our group and others have carried out a large body of work focusing on structure-activity relationships (SARs) studies of GA around its unique chemical architecture, a “caged-xanthone”^23^,^24^,^25^,^26^,^27^,^28^. Through great efforts, the simplification and modification of GA has been fully explored. As previously reported, some caged scaffolds with different rings do not have activity^25^. Additionally, changes in the orientation and spacing of the B ring or breaking its rigid planar structure will decreases its antitumor activity. As a result, the intact BCD ring with its unique caged scaffold, as shown in Fig. 1, is the minimum pharmacophore motif essential for antitumor activity^27^. Moreover, the C-9,10-α,β-unsaturated moiety in the D caged ring is also important to maintain antitumor activity^26^,^27^. It is worth noting that simplified caged xanthone analogues showed better antitumor activity and druggability compared to GA (Fig. 1).

Our group described a series of caged-xanthone-derived compounds with potent antitumor activity. **DDO-6101**, which has a simplified structure, has retained the activity of GA against various tumour cells^27^. Derivatives were designed based on **DDO-6101** to improve its *in vivo* antitumor activity, and the biological results indicated that modifications at the C(2), C(3) and C(4) sites of the B ring and the C(19) and O(16) sites of the D caged ring are well tolerated. Among these derivatives, **DDO-6267**, which has a modification at C(19), had better oral antitumor activity than GA^27^. Moreover, **DDO-6306** inhibited 52.6% of tumour growth in Heps-transplanted mice following its intravenous (IV) administration and is more potent than **DDO-6101**^26^. In addition, structure-property relationship (SPR) studies based on **DDO-6101** demonstrated that hydrophilic heteroatom-containing groups, such as those in **DDO-6306**, help enhance drug-like properties and improve antitumor activity *in vivo*, suggesting that these compounds with simplified structures are potential anticancer compounds. However, their limited solubility and drug-like properties still limit their clinical application. From these diverse caged-xanthone-derivatives, the effect of the group at the C(1) site of **DDO-6101** has not been explored thoroughly. Therefore, to explore the C(1) site, hydrophilic heteroatom-containing groups at the C(1) site were introduced with the aim of developing more potent compounds, completing the SAR and SPR studies of the caged-xanthones, and exploring this region to determine whether modifying it can improve the antitumor potency and drug-like properties of the compounds. Here, we describe the design, synthesis and evaluation of novel caged-xanthone-derivatives of **DDO-6101** to develop novel xanthones as anticancer agents.

## Results and Discussion

### Compound Design and Synthesis

**Figure 2:**
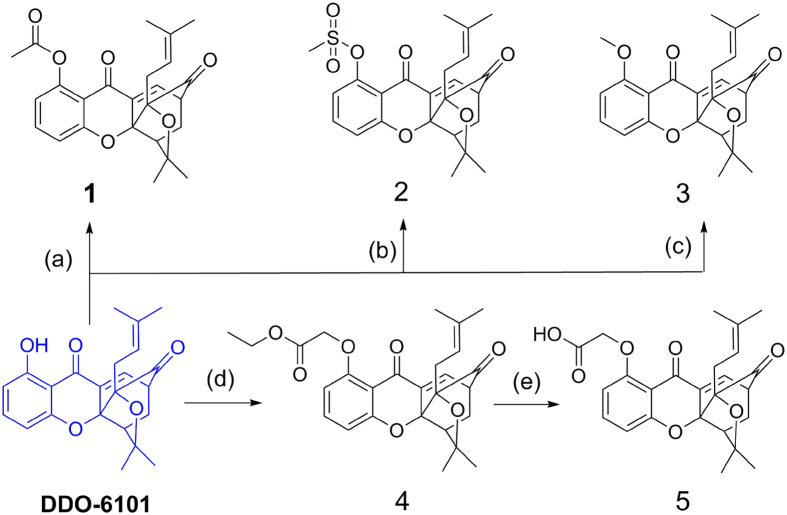
*Reagents and conditions:* (**a**) Ac_2_O (1.5 equiv.), 4-dimethylaminopyridine (DMAP; 1.5 equiv.), dichloromethane (DCM), room temperature (rt), 5 h, 99%; (**b**) CH_3_SO_2_Cl (1.5 equiv.), triethanolamine (TEA, 1.5 equiv.), DCM, rt, 5 h, 97%; (**c**) MeI (1.5 equiv.), K_2_CO_3_ (1.5 equiv.), acetone, rt, overnight, 99%; (**d**) BrCH_2_CO_2_Et (1.2 equiv.), K_2_CO_3_ (1.2 equiv.), dimethylformamide (DMF), 45 °C, 1 h, 92%; (**e**) 17% HCl, tetrahydrofuran (THF), rt, 12 h, 85%.

**DDO-6101** contains a phenolic hydroxy group that is a potential modification site. First, derivatives **1**–**5** were designed to explore the influence of some ester and alkyl substituent groups at the C(1) site. The designed cage xanthone derivatives **1**–**5** and synthetic routes are shown in Fig. 2. The starting material **DDO-6101** was generated using previously described chemical pathways^27^. Acylation of **DDO-6101** in the presence of DMAP and acetic anhydride produced caged xanthone **1**. Methyl sulphonylation of **DDO-6101** with methylsulfonyl chloride and triethylamine produced **2**. **DDO-6101** was methoxylated by methyl iodide and potassium carbonate at RT. **4** was prepared using bromoacetic acid ethyl ester and potassium carbonate in a polar aprotic solvent (DMF). The hydrolysis of **4** in diluted hydrochloric acid and tetrahydrofuran yielded **5**. The caged xanthone was not stable in an alkaline environment, and thus, hydrolysis was performed under acidic conditions.

Then, the antiproliferative activities of the derivatives were assessed, as shown in Table 1. Doxorubicin was used as the positive control for the *in vitro* assay. **1** (acetylation of C1) exhibited inhibitory activity toward the three cancer cell lines, similar to that of **DDO-6101**; **2** (methylsulphinyloxy group) showed slightly decreased activity compared to **DDO-6101**; and **3** (methoxy group) was approximately 4–8-fold less active. The presence of electron-withdrawing substituents on C1 improved the activity of the compounds, whereas electron-donating groups on C1 inhibited cytotoxicity. Preliminary SAR studies indicated that the *α*,*β*-unsaturated ketone moiety of the CD caged region in **GA** was the key motif to maintain its antitumor activity. A substituent group on C1 substantially influenced the electrophilicity of the *α*,*β*-unsaturated double bond, and an electron-withdrawing group decreased its electron cloud density, thereby contributing to the Michael reaction with nucleophilic groups of target proteins, such as sulfhydryl and amino groups, ultimately leading to better cytotoxicity.

Compared to **3**, compound **4**, which has a longer side chain, exhibited reduced activity, indicating that extending the substituent carbon chain results in a loss of activity. The activity of **5** was far lower than that of **4**, which suggests that the presence of strong hydrophilic groups, such as carboxyl groups, causes a complete loss of cytotoxicity.

**1** showed low micromolar activity, with IC_50_ values ranging from 0.97 to 1.49 μM, comparable to those of **DDO-6101** and GA. However, compounds **2**–**5** were less cytotoxic against the three cancer lines. Therefore, caged-xanthone-derivatives were designed and prepared based on **1**.

**Figure 3:**
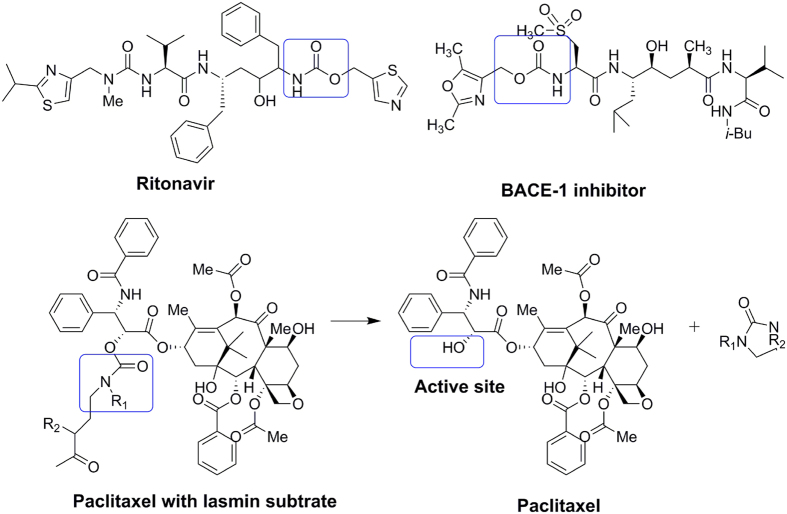
Examples of carbamate drugs and alcohol carbamate prodrugs.

As a structural motif, organic carbamate plays an important role in drug design and medicinal chemistry^29^. Because of their chemical stability and ability to permeate cell membranes, many approved drugs and clinical candidate compounds contain carbamate structures, such as Ritonavir and the BACE1 inhibitor, as shown in Fig. 3 30,31. Ritonavir, a first-generation protease inhibitor, shows excellent pharmacokinetic properties, possibly because of the increased stability of the thiazole groups with the carbonate linker^32^. Scherren *et al.* explored the characteristics of paclitaxel-2′-carbamate, which is more stable than esters and carbonates *in vivo*^33^. Thus, it was inspiring to use organic carbamate as a structural motif in further studies.

Previous modifications to **DDO-6101** revealed some SARs, and these studies identified analogues that exhibited comparable inhibitory activity. These studies showed that an OH group at the C(1) site was not required for antiproliferative activity because substituting this group with an acetyl group did not change the potency of the compound. Whether modifying this region with carbamate would increase its inhibitory activity or improve its drug-like properties was further explored.

**Figure 4:**
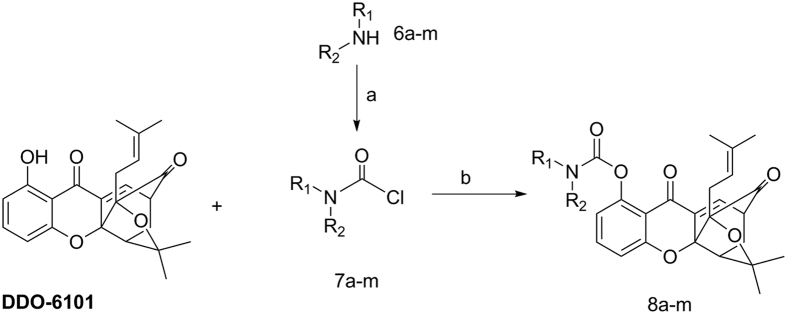
*Reagents and conditions:* (**a**) bis(trichloromethyl) carbonate (BTC, 1 equiv.), TEA (3 equiv.), DCM, 90%; (**b**) K_2_CO3 (1 equiv.), DMAP(0.1 equiv), DCM, rt, 3 h, 60–90%.

Using excess carbamyl chloride with a potassium carbonate base and DMAP catalyst, **DDO-6101** can be converted into **8a–8m**, with yields of 50–70%, as shown in Fig. 4. Carbamyl chloride can be obtained using different reactions with triphosgene. The chemical structures of these derivatives were inferred using ^1^H-nuclear magnetic resonance (NMR), ^13^C-NMR, electron ionization mass spectrometry (EI-MS), and infrared (IR) spectroscopy. All of the synthesized compounds yielded acceptable elemental analyses and high-resolution EI-MS (HREI-MS) data.

### *In vitro* Cytotoxic Effects

The antiproliferative activities of the 15 synthesized caged xanthone derivatives, lead compound **DDO-6101**, **GA** and doxorubicin were assessed using a tetrazolium-based colorimetric (MTT) assay and human hepatocellular carcinoma cell line (HepG2), human colon cancer cell line (Hct116) and human breast cancer cell line (MDA-MB-231), as previously reported. The antiproliferative activities, expressed as IC_50_ values, are summarized in Table 2.

In general, most of the caged xanthones exhibited potent antiproliferative activities against HepG2, HCT116, MDA-MB-231 and A549 cells, with IC_50_ values in the low micromolar range, which are comparable to those of GA and **DDO-6101**. The results indicated that these alkyl carbamate derivatives function in the cell.

However, different compounds showed different activities against the three cell lines. **8e**, **8f** and **8l** showed selective activity against HepG2 cells, with growth inhibition IC_50_ values of 0.99 ± 0.01, 0.95 ± 0.09 and 0.34 ± 0.27 μM, respectively. The HCT116 cell line was sensitive to **8d**, **8e**, **8f** and **8i**, with IC_50_ values of 0.71 ± 0.04, 1.06 ± 0.09, 1.02 ± 0.07 and 1.08 ± 0.02 μM, respectively. The MDA-MB-231 cell line was sensitive to **8a**, **8c**, **8m** and **8o**, with IC_50_ values of 0.17 ± 0.04, 0.25 ± 0.12, 0.76 ± 0.09 and 0.66 ± 0.02 μM, respectively. The A549 cells were less sensitive to all of the tested compounds compared to the other cell lines. However, these compounds showed better cytotoxic activity against Taxol-resistant and cisplatin-resistant A549 cell lines. In particular, **8l** displayed 10–15-fold higher activity against the resistant A549 cell line, but was less active against A549 cells.

Based on Table 2, introduction of carbamate with long aliphatic side chains, as in **8a–8c**, **8j** and **8k**, resulted in slightly lower activity than those of pentacyclic or hexacyclic compounds, as in **8d–8i**, **8l–8m**. Few differences in cytotoxicity were found between pentacyclic and hexacyclic compounds. Among the series of caged derivatives, **8l** showed the most potent inhibitory activity against HepG2, HCT116, and MDA-MB-231 cells *in vitro*, with IC_50_ values of 0.34 ± 0.27, 2.49 ± 0.33 and 0.88 ± 0.5 μM, respectively. Notably, **8l** was more active *in vitro* than the lead compound **DDO-6101**.

### Structure-property Relationship (SPR) Studies

The physicochemical characteristics of compounds directly determine their potential drug-like properties. Assessing a compound’s physicochemical properties before performing *in vivo* evaluations can guide further studies and minimize the time and cost associated with drug discovery. Therefore, parallel to the *in vivo* study, these solubilities of these compounds in water were experimentally measured using high-performance liquid chromatography (HPLC). Based on the results of full wave scanning, 290 nm was chosen as the maximum absorption wavelength for HPLC detection. However, no improvement in water solubility was observed for non-carbamate-containing compounds^1^,^2^,^3^,^4^,^5^ compared to **DDO-6101** and GA, as shown in Table 3. Modifying the compounds substantially improved their water solubility when the lipophilic tail was linked with the pharmacophore on C(1), particularly when the primary pharmacophore was an amide. To improve the water solubility by a large margin, some compounds were generated via salification with HCl, such as **8n** and **8o**, which exhibited water solubility exceeding 40 mM (Table 3).

**Figure 5:**
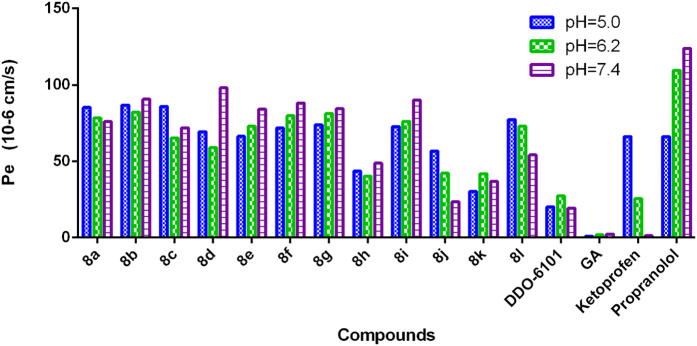
Experimental determination of the membrane permeability of selected compounds at different pH values.

Permeability is another important property that reflects the ability of molecules to diffuse through cell membranes. The permeability coefficients were determined using a standard parallel artificial membrane permeability assay (PAMPA) on a PAMPA Explorer instrument (Pion, Inc., MA, USA)^34^. As shown in Fig. 5, the introduction of hydrophilic substituents, such as in **8b**, **8d**, **8i** and **8l**, improved the permeability of the compounds relative to **DDO-6101** and **GA**, suggesting that modification with the carbamate moiety could enhance permeability.

### 8n Induces Tumour Cell Apoptosis

**Figure 6:**
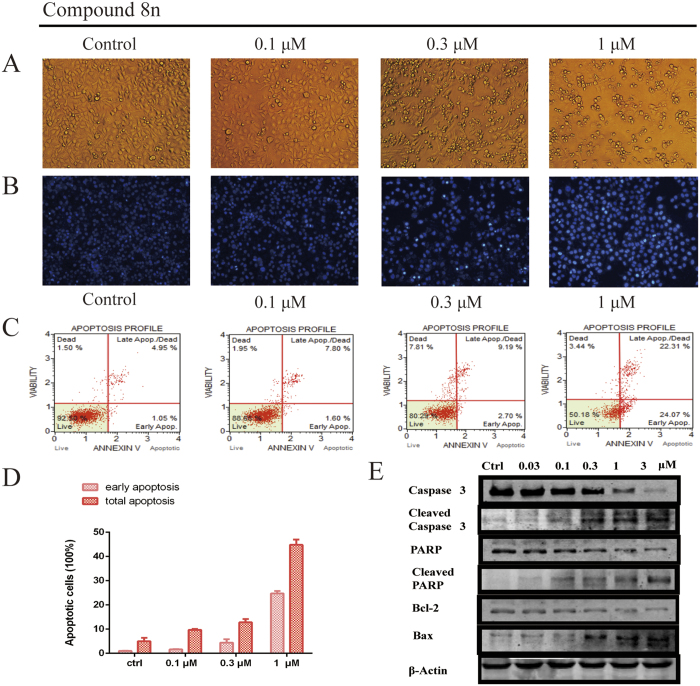
(**A**) Morphological changes in cancer cells and nuclei. (**B**) **8n** induced apoptosis in HepG2 cells. Cultured HepG2 cells were treated with the indicated concentrations of **8n** for 48 h. The cells were then were stained with DAPI and observed by fluorescence microscopy. (**C**) **8n** induces apoptosis in a dose-dependent manner. HepG2 cells were treated with different concentrations of **8n** for 48 h, and Annexin V and propidium iodide staining were performed. (**D**) The percentages of early apoptotic and total apoptotic cells were quantified using flow cytometry. *p < 0.05; **p < 0.01. (**E**) The expression of apoptotic-related proteins in HepG2 cells treated with **8n**. HepG2 cells were treated with various concentrations of **8n** for 24 h and were then harvested and lysed. The expression levels of Caspase-3, Cleaved Caspase-3, Bax, Bcl-2, PARP and cleaved PARP were measured by Western blotting.

Because it has superior cytotoxicity and physicochemical properties, **8n** was chosen as a representative compound to determine whether caged xanthone derivatives modified with carbamate induced tumour cell apoptosis as well as **GA**. First, the influence of **8n** on the cytoskeleton was evaluated using morphological observations. After treatment with **8n**, the morphological features of tumour cells changed greatly, and the changed characteristics included the rounding-up of cells, rough membrane surfaces, and more spherical or ovoid cytoplasmic fragments (Fig. 6A). However, the control group, which lacked compound **8n**, had good growth. Next, we examined whether the morphological changes were the result of apoptosis induced by compound **8n** using DAPI staining and FACScan. DAPI-stained nuclei indicated that there were condensed chromatin and nuclear fragmentation in the **8n** treatment group, whereas there were little nuclear condensation and apoptotic bodies (Fig. 6B). Similar findings were observed with Annexin-V-PI staining, which showed that compound **8n** induced apoptosis in HepG2 cells in a dose-dependent manner, and indeed, treatment with 0.3 and 1 μM **8n** for 48 h induced apoptosis in more than 11.89% and 46.3% of cells, respectively (Fig. 6D).

To corroborate these results, the effects of **8n** on the cleavage of Caspase-3 and poly(ADP-ribose) polymerase (PARP), two well-established biochemical markers of apoptosis, were determined. Treatment with **8n** increased the levels of cleaved caspase-3/PARP and decreased the amount of full-length Caspase-3/PARP. The apoptosis inducers Bcl-2 and Bax were also affected by **8n**, suggesting that **8n** led to apoptosis via the mitochondrial apoptosis pathway (Fig. 6E).

### 8n exhibits potent *in vivo* activity in hepatoblastoma xenograft models

**Figure 7:**
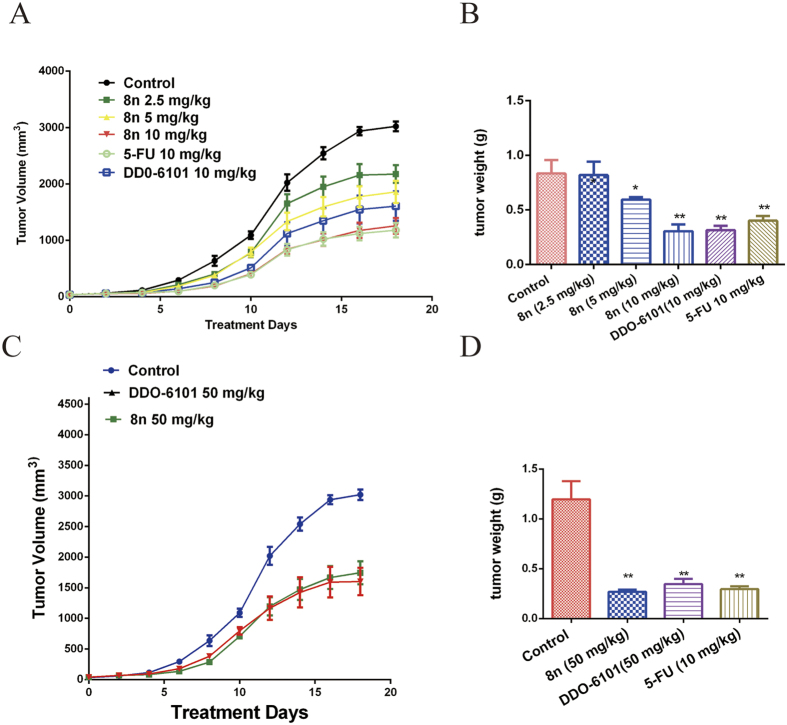
(**A**) The tumour diameters were measured and used to calculate the tumour volumes. The HepG2 xenograft-bearing mice were intravenously injected with 2.5, 5, 10 and 50 mg/kg **8n** for 20 days. (**B**) After 20 days, mice were sacrificed and the individual tumours were weighed. *p < 0.05, *p < 0.01; Student’s t-test (n = 6). (**C**) The tumour diameters were measured and used to calculate the tumour volumes. The HepG2 xenograft-bearing mice were orally administered 50 mg/kg **8n**, 50 mg/kg **DDO-6101** or 10 mg/kg **5-FU** for 20 days.

Given the *in vitro* anti-tumour cell activity of **8n**, we continued to evaluate its *in vivo* effect in hepatoblastoma xenograft models using previously reported methods^35^, and the water-soluble anti-cancer drug 5-fluorouracil (**5-FU**) was used as a positive control. After tumours were established, the mice were IV injected with 2.5, 5, 10 or 50 mg/kg **8n;** the lead compound **DDO-6101** and antitumour drug **5-FU** were used as controls. All mice had normal weights and quality of life in *in vivo* tests. As shown in Fig. 5A,B, twice-daily treatment with 2.5, 5 and 10 mg/kg **8n** produced significant inhibition of the tumour volume by 27.97%, 38.50%, and 48.36%, respectively, compared with the **5-FU** group (50.95%) and the **DDO-6101** group (36.81%). **8n** displayed a significant and dose-dependent inhibitory effect on the growth of HepG2 tumours in nude mice. The *in vivo* activity of **8n** was also evaluated after oral administration. When administered daily at a dosage of 50 mg/kg, **8n** inhibited tumour growth by 30.12%, suggesting that **8n** has good bioavailability (Fig. 7C,D). Thus, compared with **DDO-6101**, the carbamate derivative **8n** exhibited better oral activity and was much more potent *in vivo*. This observation may be attributed to the improved physicochemical properties of **8n** compared to those of **DDO-6101**, such as aqueous solubility and cell membrane permeability, which were provided by modification with the carbamate moiety. Taken together, the results showed that **8n** inhibited tumour growth *in vivo*.

### The *In Vitro* and *In Vivo* Anti-Angiogenesis Activities of the Caged Xanthone Derivative 8n

**Figure 8:**
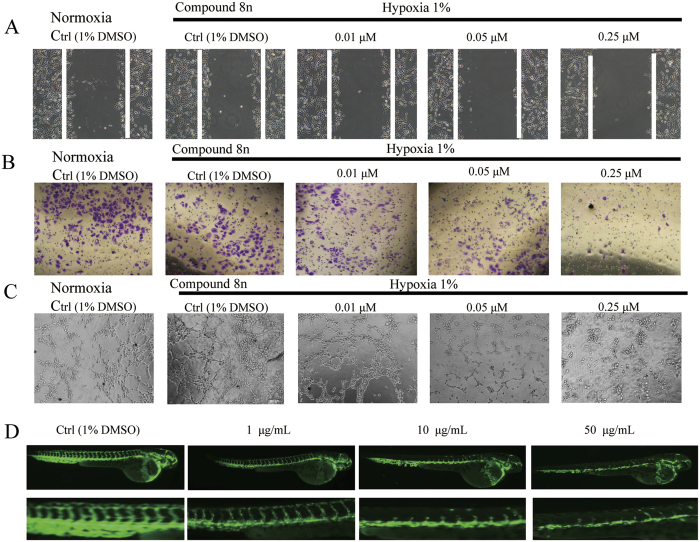
8n had anti-angiogenesis activity *in vitro* and *in vivo*. (**A**) A wound-healing assay was used to evaluate the motility of HUVECs after treatment with **8n** for 24 h. (**B**) Changes in cell motility were evaluated using a transwell assay of cell migration and invasion. (**C**) The effect of **8n** on tube formation by HUVECs. HUVECs were incubated with **8n** for 6 h and then transferred to Matrigel for 6 h. (**D**) Zebrafish embryos treated with blank control or 1, 10, or 50 μg/ml of **8n**.

**GA** was reported to inhibit tumour angiogenesis and may be a viable drug candidate for anti-angiogenesis and anticancer therapies^15^. Therefore, the anti-angiogenesis activity of the caged xanthone derivative **8n** was evaluated. Human umbilical vein endothelial cells (HUVECs) were used in all experiments. Cell migration is critical for endothelial cells to form blood vessels during angiogenesis, tumour growth and metastasis. **8n** suppressed hypoxia-induced migration in a concentration-dependent manner at 0.01, 0.05 and 0.25 μM compared to the controls (Fig. 8A). Endothelial cell invasion is a critical part of angiogenesis; therefore, a transwell assay was performed to evaluate the ability of inactivated HUVECs to pass through the transwell membrane barrier in the presence of **8n**. As shown in Fig. 8B, 0.25 μM **8n** almost completely inhibited the invasion of inactivated HUVECs, suggesting that **8n** can significantly inhibit the invasion properties of endothelial cells. In the tube-formation assay (Fig. 8C), medium from HepG2 cells treated with **8n** significantly impaired the tube-forming ability of HUVECs compared to that from the controls (untreated HepG2 cells or medium alone).

Zebrafish has been proposed as a valid alternative animal model for investigating angiogenesis^35^,^36^,^37^. The effect of **8n** on embryonic angiogenesis in zebrafish was evaluated. Figure 8D shows the results of treating zebrafish embryos with **8n** or the vehicle control. Treating live fish embryos with 50 μg/ml **8n** completely blocked the formation of intersegmental vessels, while preserving fluorescence in the dorsal aorta and major cranial vessels. At a dose of 10 μg/ml or 5 μg/ml **8n**, the formation of intersegmental vessels was considerably inhibited compared with the blank control group, indicating a dose-dependent inhibition pattern. Together, these results indicated that **8n**, a caged-xanthone-derivative of GA with a carbamate moiety, exhibited antiangiogenic effects *in vitro* and *in vivo*.

### The Caged Xanthone Derivatives Showed Anti-Angiogenesis Activity by Targeting Hsp90 and HIF-1α

**Figure 9:**
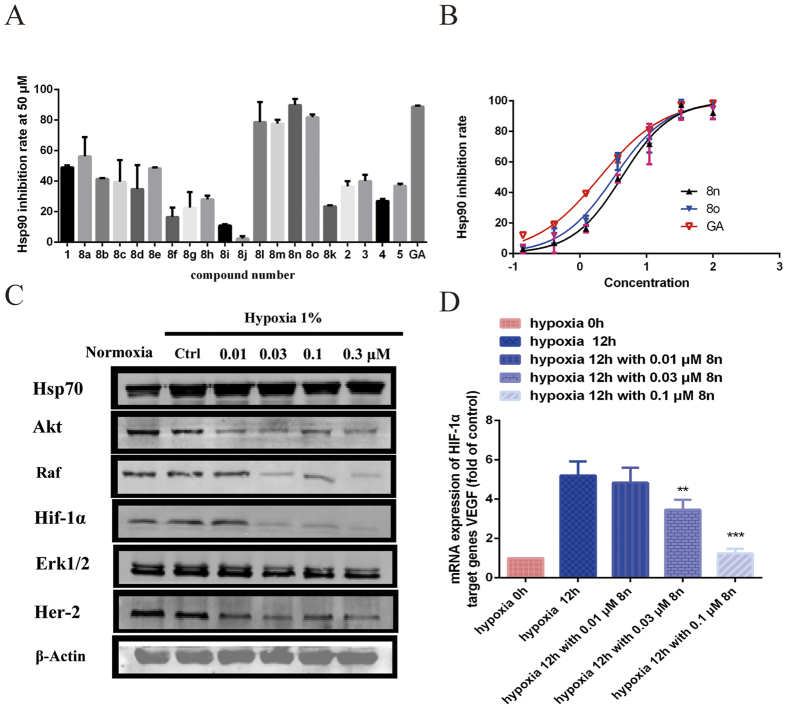
(**A**) The caged compounds (50 μM) inhibited the Hsp90 ATPase activity in a HTRF-based inhibition assay. (**B**) The IC_50_ values for the inhibition of Hsp90 ATPase by **8n**, **8o** and GA as detected by the HTRF Transcreener ADP kit. The test compounds were diluted to obtain a range of concentrations and incubated with Hsp90 and ATP. The amount of ADP generated was detected. (**C**) Western blots of the Hsp90 client proteins after HepG2 cells were treated with the indicated concentrations of **8n** for 24 h. (**D**) The mRNA expression of VEGF was determined by real-time quantitative reverse-transcriptase polymerase chain reaction (RT-PCR) and analysed by the delta Ct method. The bars represent the means with standard error of the mean (SEM) and the fold change in the VEGF/GAPDH ratio relative to the vehicle-treated group. The different significance levels are indicated as **P < 0.05.

Lianru Zhang, Brian S. J. Blagg, *et al.* reported that GA inhibited Hsp90 ATPase *in vitro*^21^,^38^. The effects of these compounds on Hsp90 ATPase activity were examined by measuring the ability of Hsp90 to hydrolyse ATP using a homogeneous time-resolved fluorescence (HTRF) assay to determine whether the caged-xanthone-derivatives of **GA** inhibited Hsp90. The activities, expressed as the inhibition ratio at 50 μM, are summarized in Fig. 9A. 17-(Dimethylaminoethylamino)-17-demethoxygeldanamycin (**17-DMAG**), an Hsp90 N-terminal inhibitor, was used as a positive control and exhibited an IC_50_ value of 0.919 μM in the Hsp90 ATPase assay. Analysing the results of the HTRF assay showed that, in general, some compounds maintained the same activity as **GA**. Among them, compounds **8n–8o** were identified as good to moderate Hsp90 inhibitors, with IC_50_ values of 4.21, 3.25 and 1.89 *μ*M, respectively (Fig. 9B). The inhibition of Hsp90 could decrease client protein levels. Based on the enzymatic, structural, and antiproliferative results, **8n** was chosen for further investigation.

The influence of **8n** on the degradation of Hsp90 client proteins was monitored by WB analysis. HepG2 cells were treated with different concentrations of **8n** for 24 h. The results showed that the expression levels of client proteins, such as HER-2, Akt and Erk1/2, were dramatically reduced after **8n** treatment, as monitored by WB (Fig. 9C). However, the expression of Hsp70 changed little, suggesting that **8n** did not induce the heat shock reaction like other Hsp90 N-terminal inhibitors. HIF-1α, as a key modulator of angiogenesis and an important client protein of Hsp90, was also examined, and the expression of HIF-1α decreased after treatment with **8n**. The hypoxic induction of the expression of vascular endothelial growth factor (VEGF), a target gene of HIF-1α, was substantially repressed in the presence of **8n** (Fig. 9D). Taken together, these results indicate that inhibiting Hsp90 results in the inhibition of HIF-1α, thereby down-regulating the transcription of pro-angiogenic cytokines and inhibiting angiogenesis.

## Conclusion

In conclusion, a small library of caged-xanthone-derivatives containing carbamate scaffolds were designed and synthesized. These carbamate derivatives were synthesized to improve the drug-like properties of the original compound. The MTT assay revealed that these compounds manifested comparable antiproliferative activities and better physicochemical properties, which contributed to improving their *in vivo* activities. The mechanisms of these compounds were also explored. **8n** (**DDO-6337**), the representative compound, showed moderate inhibitory activity toward Hsp90 ATPase and resulted in the degradation of Hsp90 client proteins, such as HIF-1α, ultimately contributing to this compound’s antitumor and anti-angiogenesis activities.

## Materials and Methods

### Compounds and Reagents

The synthetic routes of target compounds and their identification results are shown in the supporting information files. The compounds used in the biological assay were in DMSO with a stock concentration of 10 mM, and the maximum concentration of DMSO used was no more than 0.1%. All of the chemical reagents and biological reagents, including DMSO, were purchased from Sigma-Aldrich (St Louis, MO, USA). All cell lines used in study were purchased from Typical Culture Preservation Commission Cell Bank, Chinese Academy of Sciences (NCB). The antibodies used from Cell Signaling Technology (Beverly, MA, USA) were the PARP antibody (9542L), cleaved-PARP antibody ^5625^, anti-Caspase 3 antibody ^9664^, cleaved caspase-3 antibody ^9664^, Bcl-2 antibody ^3498^, Bax antibody ^2772^, c-RAF antibody ^9422^, Akt antibody (9272S), Hsp70 antibody ^4876^, Erk1/2 antibody ^4370^, and HER-2/ErbB2 antibody ^2242^. The HIF-1a antibody (ab51608) was purchased from Abcam (Cambridge, MA, USA). The β-actin antibody (60008-1-Ig) was from the Proteintech Group (Wuhan Sanying Biotechnology, Hubei, China). Cisbio Bioassays in France supplied the HTRF transcreener ADP kit. Adriamycin (S1208) was purchased from Selleck (Texas, USA).

### Animals

All animal studies were conducted in compliance with the Guide of Chinese Academy of Medical Sciences and under the guidance of the Animal Ethics Committees of the Institute of Materia Medica and China Pharmaceutical University. Female nude mice at an age of five to six weeks with body weights ranging from 18 to 22 g were obtained from the Shanghai Slac Laboratory Animal Limited Company. The mice were maintained in a sterile environment at 21 °C to 25 °C and a relative humidity of 55 ± 5%. Autoclaved deionized water and irradiated pelleted food as well as a 12 h light/12 h dark cycle were provided during the whole experimental period. The mice were maintained under the guidelines of the National Science Council of the People’s Republic of China.

### Cell Proliferation Assay

The inhibition of tumour cell growth was measured by a modified tetrazolium (MTT) salt assay, as previously described^39^. Cells were cultured in a 96-well plate and treated with culture medium alone or different concentrations of the compounds. The cells were incubated for 72 h, and then, the MTT assay reagent was added. After a 4 h incubation, the formazan product was quantitated at 570 nm. The IC_50_ values (the concentrations that gave rise to 50% inhibition of cell viability) were calculated using GraphPad Prism 6 using a variable slope (four parameters).

### Hsp90 ATPase Activity

To measure the Hsp90 ATPase activity, a ADP Hunter Plus Assay kit from DiscoverX was used as previously described^39^. Recombinant human Hsp90 protein (200 nM/L, Stressgen) and compounds at different concentrations were pre-incubated in a 384-well black microplate at 37 °C for 0.5 h in the presence of assay buffer from the kit. Then, the plate was incubated for another 0.5 h after the addition of ATP (100 μM), followed by addition of the detection reagent, and the fluorescence was measured at an excitation wavelength 540 nm and emission wavelength of 620 nm using a spectrophotometer (Varioskan multimode, Thermo). The IC_50_ was obtained using the GraphPad Prism software, version 6.0.

### Western Blotting

The cells were washed with PBS and lysed with ice-cold lysis buffer supplemented with protease inhibitors (Roche) for 30 min. The protein content of the supernatants of lysates was determined using a bicinchoninic acid assay. Equal 50 μg total protein samples were separated by sodium dodecyl sulphate polyacrylamide gel electrophoresis (SDS−PAGE) and then transferred onto PVDF membranes (Millipore) at 90 volts for 90 mins. After blocking the membrane with 5% skim milk for 1 h at RT, the membranes were incubated with the primary antibody overnight at 4 °C. Then, the membranes were incubated with a DyLight 800-labeled secondary antibody in the dark for 1 h. Detection of specific proteins was performed with the Odyssey infrared imaging system (LI-COR, Lincoln, Nebraska, USA).

### Transwell Migration Assay

A transwell migration assay was performed as previously described with minor modifications^15^. HUVECs (4×10^4^ cells per well) with compound **8n** were seeded in the top of transwells (Corning Incorporated) and allowed to migrate for 4 h at 37 °C, 5% CO_2_ and 1% O_2_. The cells on the top surface of the Matrigel were removed using a cotton swab, and the migrant cells were fixed for 30 min with 4% (wt/vol) formaldehyde. The cells were then washed three times with PBS, coloured with 0.1% crystal violet, and microphotographed. Each assay was performed three times using triplicate samples.

### Tube Formation Assay

The tube formation assay was performed as previously reported with some modifications^40^. A 48-well cell culture plate and the tips used were pre-chilled at −20 °C for 10 mins and then carefully coated with Matrigel (200 μL/well; Becton Dickinson, Bedford, MA, USA). Caution was taken to avoid bubbles. The plate was cultured at 37 °C for 30 minutes, and then, the Matrigel became solid. HUVECs were treated with **8n** for 4 h. The cells were collected and suspended in serum-free medium. Then, 2 ×10^4^ cells were seeded onto the Matrigel under normoxic or hypoxic conditions. After 6–8 h, photos were taken of the tube formed by HUVECs in five random fields per well.

### Wound Healing Assay

HUVECs were incubated in a 6-well plate until full confluence. Monolayers of HUVECs (90% confluent) were starved in serum-free medium for 12 h and carefully scratched using a 200 μl sterile pipette tip. The debris was washed twice with PBS. Then, the HUVECs were treated with **8n** or the vehicle in full medium. Cells were cultured under hypoxic conditions for 24 h. Then, the wound edges were photographed under an inverted-phase microscope and measured.

### Cell Death Analyses

HepG2 cells were grown on glass coverslips and fixed in 4% paraformaldehyde. Then, the nuclei were stained with DAPI (Sigma Aldrich) and visualized to evaluate the morphological evidence of apoptosis. **8n**-induced apoptosis was assessed using a fluorescence microscope and the Annexin V-FITC apoptosis Detection Kit (Beyotime Biotechnology, Shanghai, China) according to the manufacturer’s instructions. Briefly, cells treated with **8n** for 48 h were harvested and washed in PBS. The cells were diluted to 1×10^6 ^cells/mL and incubated with Annexin V and propidium iodide (PI) for 10 minutes in binding buffer in the dark at RT. Stained cells were analysed with a FACScan analyser (argon laser, Becton Dickinson, USA). All data were analysed by using the FlowJo software.

### *In vivo* Fluorescent Zebrafish Assay

Transgenic zebrafish (VEGFR2: GFP) embryos were supplied using the drug screening platform of the Shandong Academy of Sciences Institute; they were grown and maintained according to the same protocols^41^. The screen was carried out in 24-well plates. Compounds were prepared initially as a 10 mg/mL stock solution in dimethyl sulfoxide (DMSO). The stock solution was diluted to the relevant assay concentration with fish water, and 0.5% DMSO served as a vehicle control. The embryos were distributed in 24-well plates, with 10 embryos placed in each well. Then, a diluted solution of each compound was added. The embryos were exposed to the compound solution and incubated at 28.5 °C. After 24 h, the zebrafish were anesthetized with 0.01% tricaine and imaged under a fluorescence microscope (Olympus, Inc.) equipped with a SZX16 microscope and a DP2-BSW digital camera (Olympus, Inc.). The animal experiments were performed in accordance with the approval of the Chinese Academy of Medical Sciences and the Biology Institute of Shandong Academy of Sciences. All studies were performed in accordance with the approved guidelines from the China Zebrafish Resource Center with the approved protocol number of 2012CB944504.

### A statement identifying the institutional and/or licensing committee experimental approval

All animal studies were performed in compliance with the Chinese Academy of Medical Sciences and the Animal Ethics Committees of the Institute of Materia Medica. The animal experiments were performed in accordance with the National Institutes of Health Guide for the Care and Use of Laboratory Animals with the approval of the Center for New Drug Evaluation and Research of China Pharmaceutical University (Nanjing, China).

### Human Tumour Xenograft Studies

All studies were performed in compliance with the relevant guidelines and regulations. Tumours on female nude mice were established by injecting 1×10^7^ HepG2 cells on the dorsal surface. After four weeks, tumours of approximately 150 mm^3^ were cut into pieces that were 3 mm×3 mm×3 mm in size and were then implanted in the dorsal surface of other female nude mice. One week later, mice implanted with xenografts of 100 mm^3^ were selected, randomized, and intraperitoneally injected with 2.5, 5, or 10-mg/kg **8n** or vehicle daily for another three weeks. The tumour dimensions were measured daily with vernier calipers, and the tumour volumes were calculated using the formula: volume = (width)^2^ × length/2.

### Determination of the Physicochemical Properties

A HPLC system with an ultraviolet (UV) detector was used to detect the solubility of the compounds. The optimal wavelength was 290 nm. Ethanol/water (85:15) was an efficient eluent at a flow rate of 0.5 mL/min. The compounds were stirred in deionized water overnight to maximize the retention of the compounds in solution. Then, the solutions were filtered and the supernatant was used as a fluid sample for HPLC detection. The permeability (Pe) of some of the compounds was tested with the help of a standard PAMPA (pION), as previously reported^34^. PAMPA was performed on a PAMPA Explorer instrument (pION, Inc., Woburn, MA) with the PAMPA Explorer command software (Version 3.7.4.1). The compounds were diluted to 10 mM with the system solution buffer (pH 7.4). Then, 150 μL of the diluted compound solution was transferred to a UV plate, and the UV spectrum was collected and used as a reference. Then, paint the membrane with 5 μL of gastrointestinal tract (GIT) lipid. The acceptor chamber was filled with 200 μL of acceptor solution buffer, and the donor chamber was filled with 200 μL of diluted compound solution. The PAMPA sandwich was assembled and left at 25 °C for 4 h. The UV spectra (240–500 nm) from the donor and the acceptor were collected. The permeability coefficient was calculated with the PAMPA Explorer Command software (Version 3.7.4.1) based on the area under the curve (AUC) of the reference plate, the donor plate, and the acceptor plate. The permeability coefficient of the compound was tested for four times, and the data are shown as the average values. Ketoprofen (4.5×10^−6 ^cm/s) and propranolol (112.8×10^−6 ^cm/s) were used as the standards in this assay.

### Statistical Analysis

All of the reported values are presented as the means ± standard deviations (SDs) from at least two independent experiments performed in triplicate. When necessary, the two-group differences were statistically compared using t-tests. All of the statistical tests were performed using GraphPad Prism 6.0.

## Additional Information

**How to cite this article**: Xu, X. *et al.* Novel Natural Product-like Caged Xanthones Bearing a Carbamate Moiety Exhibit Antitumor Potency and Anti-Angiogenesis Activity *In vivo.* *Sci. Rep.* **6**, 35771; doi: 10.1038/srep35771 (2016).

## Figures and Tables

**Figure 1:**
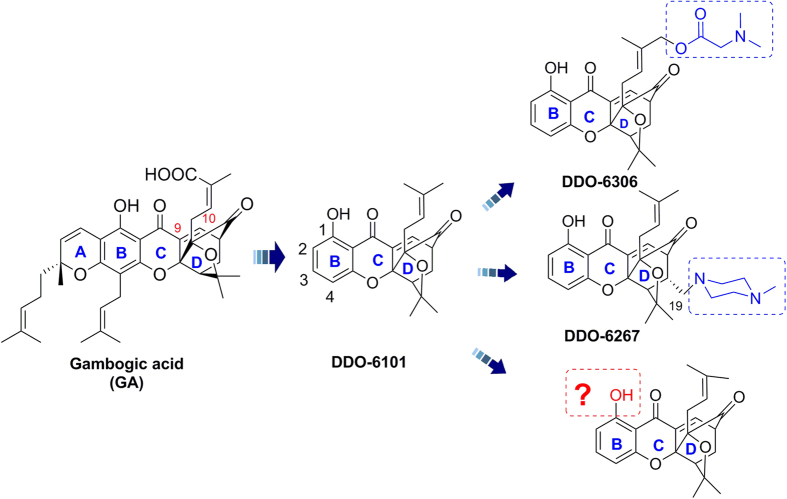
Structures of GA and its simplified caged xanthone analogs.

**Table 1:** The *in vitro* antiproliferative activities of the caged compounds based on the preliminary SAR studies.

Compound	IC_50_ (μM)		
	HepG2	HCT116	MDA-MB-231
1	1.07 ± 0.09	1.49 ± 0.17	0.97 ± 0.09
2	1.41 ± 0.06	2.12 ± 0.14	2.00 ± 0.07
3	8.55 ± 0.43	6.10 ± 0.19	1.80 ± 0.06
4	12.70 ± 0.97	8.13 ± 0.73	5.42 ± 0.57
5	>10	>10	>10
DDO-6101	1.14 ± 0.09	0.71 ± 0.1	0.32 ± 0.09
GA	2.08 ± 0.07	0.34 ± 0.08	1.50 ± 0.05
Adriamycin	0.28 ± 0.01	0.40 ± 0.04	0.49 ± 0.05

**Table 2:**
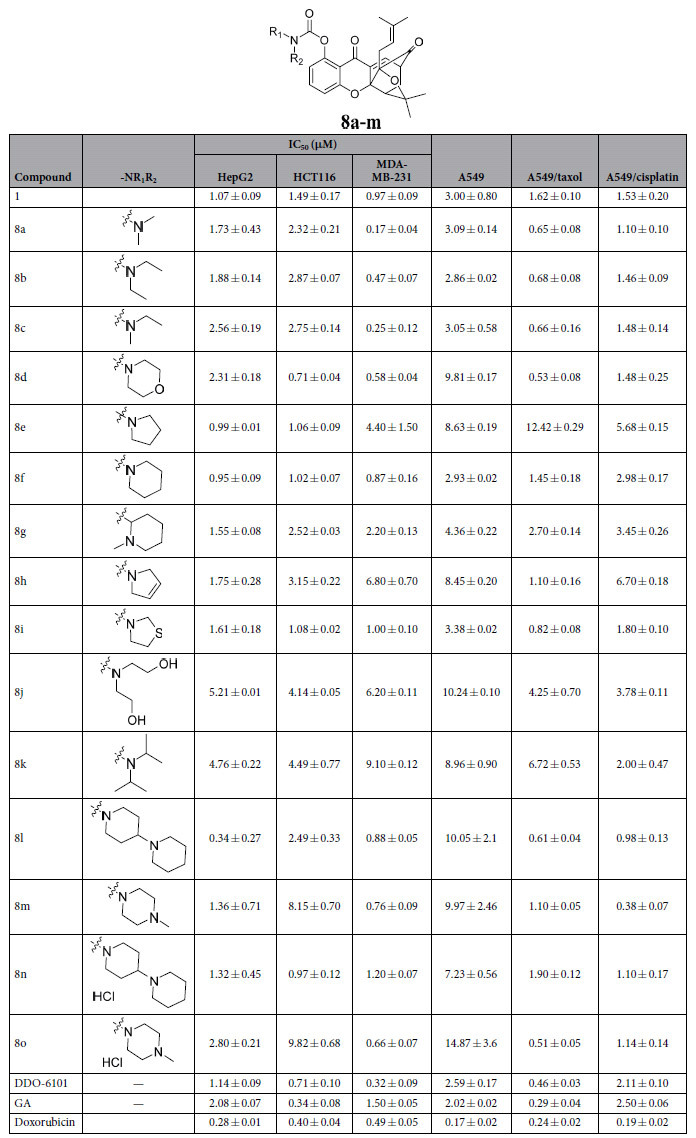
The *in vitro* antiproliferative activities of the caged compounds according to the preliminary SAR studies.

**Table 3:** The water solubility of the tested compounds probed with HPLC.

Compound	S (mM)	Compd	S (mM)
1	0.02	8g	0.02
2	0.02	8h	0.02
3	0.01	8i	0.02
4	0.01	8j	0.06
5	0.03	8k	0.03
8a	0.06	8l	0.08
8b	0.03	8m	0.64
8c	0.03	8n	>40
8d	0.04	8o	>40
8e	0.04	GA	<0.005
8f	0.05	DDO-6101	<0.005
